# Potential of Endophytic *Diaporthe* sp. as a New Source of Bioactive Compounds

**DOI:** 10.4014/jmb.2005.05012

**Published:** 2020-06-24

**Authors:** Kashvintha Nagarajan, Woei-Yenn Tong, Chean-Ring Leong, Wen-Nee Tan

**Affiliations:** 1 Chemistry Section, School of Distance Education, Universiti Sains Malaysia, 11800 Minden, Penang, Malaysia; 2 Drug Discovery and Delivery Research Laboratory, Universiti Kuala Lumpur, Malaysian Institute of Chemical and Bioengineering Technology, 78000 Alor Gajah, Melaka, Malaysia

**Keywords:** Endophytes, *Diaporthe* sp., *Phomopsis* sp., bioactive compounds, bioactive natural products, drug discovery

## Abstract

Endophytic fungi are symbiotically related to plants and spend most of their life cycle within them. In nature, they have a crucial role in plant micro-ecosystem. They are harnessed for their bioactive compounds to counter human health problems and diseases. Endophytic *Diaporthe* sp. is a widely distributed fungal genus that has garnered much interest within the scientific community. A substantial number of secondary metabolites have been detected from *Diaporthe* sp. inhabited in various plants. As such, this minireview highlights the potential of *Diaporthe* sp. as a rich source of bioactive compounds by emphasizing on their diverse chemical entities and potent biological properties. The bioactive compounds produced are of significant importance to act as new lead compounds for drug discovery and development.

## Introduction

Endophytic fungi are microorganisms that inhabit the living tissues of plants without posing any harmful or deleterious symptoms to them. Often, plants accommodate one or many endophytic fungi [1]. The interaction between endophytic fungi and their hosts is mutual, as the hosts supply habitation and nutrients while the fungi secrete functional metabolites for plant growth and survival [2]. Metabolites produced by endophytic fungi have been shown to possess structural diversity and exhibited a broad range of biological activities [1, 3]. These metabolites, at some point, are similar to those produced by the host plants; indicating the potential of endophytic fungi as an alternative source of bioactive compounds [1]. Hence, it is not surprising that endophytic fungi have attracted considerable attention in producing novel bioactive compounds for exploitation in medicine, agriculture and modern industries [4].

Among the vast endophytic fungi, genus *Diaporthe* (anamorph of *Phomopsis*) is known for its strong biosynthetic capability to produce bioactive metabolites [3, 5]. *Diaporthe* sp. is a widely distributed fungal genus and colonizes a broad range of hosts. It consists of approximately 800 species, with more than 950 species attributed to *Phomopsis* sp. [6]. It is commonly isolated from above-ground plants, particularly from tropical and temperate woody plants [7]. Secondary metabolites isolated from *Diaporthe* sp. have displayed a broad spectrum of biological activities with diverse chemical entities [8, 9]. As such, this minireview summarizes the bioactive compounds produced from *Diaporthe* sp. (duration 2015-2020 February) that had colonized within different host plants, particularly highlighting on their unique chemical entities. The bioactive compounds discovered in *Diaporthe* sp. are reported within the interest of biological context, and thereby, their potential as therapeutic agents.

## Bioactive compounds from *Diaporthe* sp.

Plant-derived fungi of *Diaporthe* have been reported to produce numerous types of compounds that exhibit a range of biological activities. These compounds offer abundant bioactive core skeletons for new medicinal lead compounds, thus contributing to the research of drug discovery and development. The unique classes of compounds are discussed in the following section. Table 1 lists the isolated compounds from *Diaporthe* sp.

## Mycoepoxydienes

Fungal mycoepoxydienes were among the bioactive compounds isolated from *Diaporthe* sp.. They are formed via polyketide pathway through the condensation of acetyl-coenzyme A (CoA) and malonyl-CoA [10]. Mycoepoxydienes have a rare oxygen-bridged cyclooctadiene that serves as the core with *α,β*-unsaturated lactone moiety [11]. In 2015, Mandavid *et al*. reported the isolation of mycoepoxydiene (**1**) from the ethyl acetate extract of *Diaporthe* sp. SNB-GSS10. The compound exhibited potent cytotoxicity against human uterine cervical carcinoma KB, human breast cancer MDA-MB-435 and human lung fibroblast MRC-5 cells with IC_50_ 7.5, 17.7 and 15.8 μM, respectively [7]. In a study on *Phomopsis* sp. PSU-H188, five mycoepoxydiene derivatives; (*E*)-1893A (**20**), 1893 B (**21**), mycoepoxydiene (**1**), 2,3-dihydromycoepoxydiene (**22**) and deacetylmycoepoxydiene (**23**) were obtained. Among them, (*E*)-1893A was isolated for the first time in fungal endophytes. Similar to other reports, mycoepoxydiene was found active against human breast cancer MCF-7 (IC_50_ 9.27 μM) and human oral cavity cancer KB (IC_50_ 14.43 μM) cells while the rest were less active [7, 12]. Based on the cytotoxic mechanisms of mycoepoxydiene derivatives, they are able to induce cancer cells apoptosis *in vivo*, thus reflecting their potential for the development of anti-cancer drugs [13].

## Cytochalasins

Cytochalasin, which has often been regarded as mycotoxin, is characterized by a substituted perhydro-isoindolone moiety joined to a macrocycle [14]. In 2017, two cytochalasin derivatives, known as cytochalasin N (**25**) and diaporthalasin (**26**), were isolated from *Phomopsis* sp. PSU-H188. Both compounds did not show any cytotoxicity on cancerous KB and MCF-7 cells, but cytochalasin N exerted a significant cytotoxic effect on non-cancerous Vero cells at 4.89 μM. Meanwhile, diaporthalasin was found to inhibit Gram-positive MRSA an *Staphylococcus aureus* (MIC 4 μg/ml), which had no cytotoxicity on non-cancerous cells [12]. In a study that assessed *Diaporthe* sp. GZU-1021, six cytochalasin derivatives were obtained. Among them, diaporthichalasins A-C (**101**-**103**) were identified as new compounds, whereas phomopsichalasin G (**104**), 21-O-deacetyl-L-696,474 (**105**) and cytochalasin,H (106) were known compounds. Although the compounds had similar cytochalasin skeleton, they displayed different inhibition on lipopolysaccharide-induced nitric oxide (LPS-induced NO) production. It was deduced that both hydroxyl (C-18) and acetyl (C-21) groups present in cytochalasin H enhanced its bioactivity [15]. Besides, cytochalasins has been found to play a crucial role in interrupting the formation of filamentous actin. They are able to modify cell motility and morphology, adherence or secretion and drug resistance. These properties are essential for the development of chemotherapeutic agents in drug resistant cancer cells [14]. Therefore, multiple chemical syntheses that focus on the various functional groups present in cytochalasins may aid in the discovery of novel bioactive compounds.

## Depsidones

On the other hand, another class of compounds known as depsidones has been found in *Diaporthe* sp.. In 2018, the discovery of diaporthols A and B (**73**-**74**) produced by *Diaporthe* sp. ECN-137 was reported by Nakashima *et al*. The compounds had tetracyclic skeletons, which mimicked the core structure of purpactin A. They were examined for their activity on transforming growth factor-β1 (TGF-β1) induced wound closure of MDA-MB-231 breast cancer cells [16]. The TGF-β1, one of the mRNAs detected in most primary breast cancers, has a significant function in apoptosis, angiogenesis and cancer progression [17]. Diaporthols A and B isolated were appeared to suppress the TGF-β1-induced wound closure at 20 μM, hence signifying their potential as tumor inhibitors [16]. Reports have also emphasized that such compounds are active inhibitors in cholesteryl ester transfer protein (CETP) and acyl-CoA:cholesterol acyltransferase (ACAT), which promote the therapeutic potential of atherosclerosis [18]. Therefore, different derivatives of depsidones indicate the potential of developing new drugs.

## Azaphilones

Azaphilones, which consist of a highly oxygenated pyranoquinone bicyclic core skeleton, has received a great deal of scientific interest recently due to their interesting structural features and promising biological activities [19]. In 2018, six new polyoxygenated chloroazaphilones (isochromophilones A-F, (**61**-**66**)) and their analogues were obtained from the ethyl acetate extract of *Diaporthe* sp. SCSIO 41011. Among the newly isolated azaphilones, isochromophilones A and B were the first described azaphilones with the absence of a carbonyl group at C-6. Upon being assessed for their cytotoxicity on ACHN, OS-RC-2 and 786-O human renal carcinoma cells; isochromophilone D displayed cytotoxicity against 786-O cells with the lowest IC_50_ (8.9 μM). In cell cycle arrest and cell apoptosis, the compound induced total apoptotic cells at 56% (48 h) and 98% (72 h) at 10 μM [20]. Apart from this, various bioactivities, such as inhibitions of glycoprotein gp120-CD4 binding, protein Grb2-SH2 and MDM2-p53 interactions, heat shock protein 90 (Hsp90) and dihydrofolate reductase, were reported for azaphilones [19]. Considering the biological diversity of azaphilones, syntheses that alter the side chains or the cyclic moieties should be weighed in to assess their structure-activity relationships, so as to explore their potential for drug discovery.

## Other Organic Compounds

Other metabolites isolated from *Diaporthe* sp. include alkaloids, terpenoids, pyranones, benzophenones, bisanthraquinones, xanthones, acid derivatives, alcohols and amides. In 2017, Cui and colleagues isolated six new alkaloids, namely diaporphasines A-D (**36**-**39**), meyeroguillines C and D (**41**-**42**) along with known meyeroguilline A (**40**), 5-deoxybostrycoidin (**43**) and fusaristatin A (**44**), from *D. phaseolorum* SKS019. They were described for the first time in *Diaporthe* sp., as containing the skeletons of chromeno[3,2-c]pyridines and isoindolinones. 5-Deoxybostrycoidin, which was initially isolated from fungus Nectria haematococca, had induced cytotoxicity against human breast cancer MDA-MB-435 and human non-small cell lung cancer NCI-H460 cells with IC_50_ of 5.32 and 6.57 μM, respectively [21, 22]. Meanwhile, a new lanostanoid, known as 19-*nor*-lanosta-5(10),6,8,24-tetraene-1*α*,3*β*,12*β*,22S-tetraol (**5**) was obtained from *Diaporthe* sp. LG23. The compound is a rare tetracyclic triterpenoid with an unusual aromatic ring B system, coupled with the loss of a common methyl group at C-19. It exhibited remarkable antibacterial activity on Gram-positive *Streptococcus pyogenes* at 0.1 μg/ml, when compared to gentamicin (10.0 μg/ml) [23]. Additionally, two rare cyclic 10-membered nonenolides, known as xylarolides A and B (**92**-**93**), were obtained from *Diaporthe* sp. isolated from *Datura inoxia* Mill. It is noteworthy to highlight that xylarolide A showed potent growth inhibition on human pancreatic cancer MIAPaCa-2 and human prostate cancer PC-3 cells with IC_50_ of 20 and 14 μM, respectively [24].

Endophytic *Diaporthe* sp. is an interesting group of microorganisms that provide a rich source of bioactive and chemically diverse compounds with medicinal potential. In this minireview, a range of rare carbon skeletons of compounds isolated from *Diaporthe* sp. is discussed. They have been reported to possess cytotoxic, antimicrobial, anti-hypercholesterolemic, anti-tuberculosis, anti-ﬁbrosis, anti-diabetic, antioxidant and anti-inflammatory properties. The discovered compounds may act as potential leads for scientists to synthesize potent analogues. It has been observed that most of the bioactivity studies of *Diaporthe* sp. are focused on in vitro. Therefore, in depth in vivo and a series of mechanism studies regarding these bioactive compounds are in need for their future application as therapeutic agents.

## Figures and Tables

**Fig. 1A:**
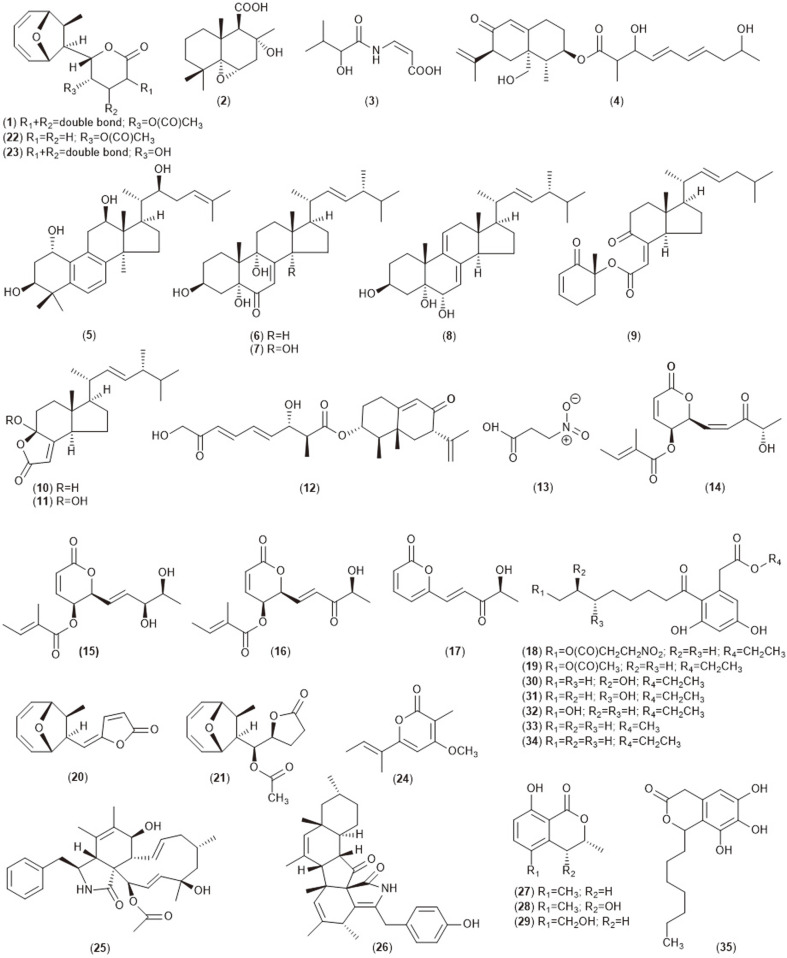
Bioactive compounds (1-35) isolated from endophytic *Diaporthe* sp.

**Fig. 1B:**
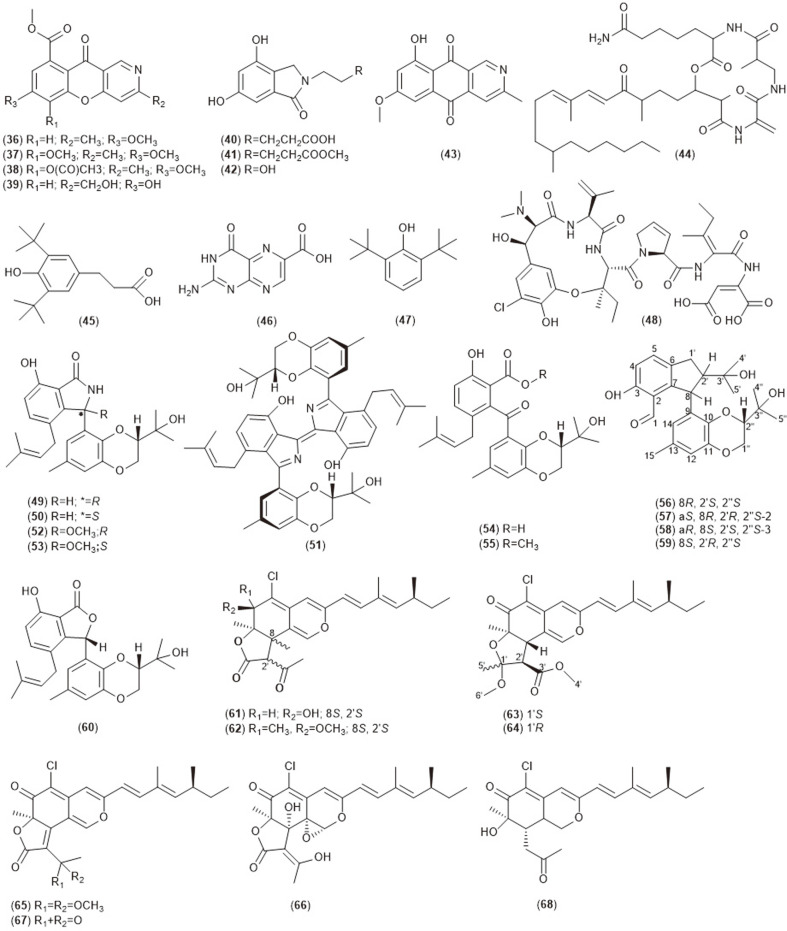
Bioactive compounds (36-68) isolated from endophytic *Diaporthe* sp.

**Fig. 1C:**
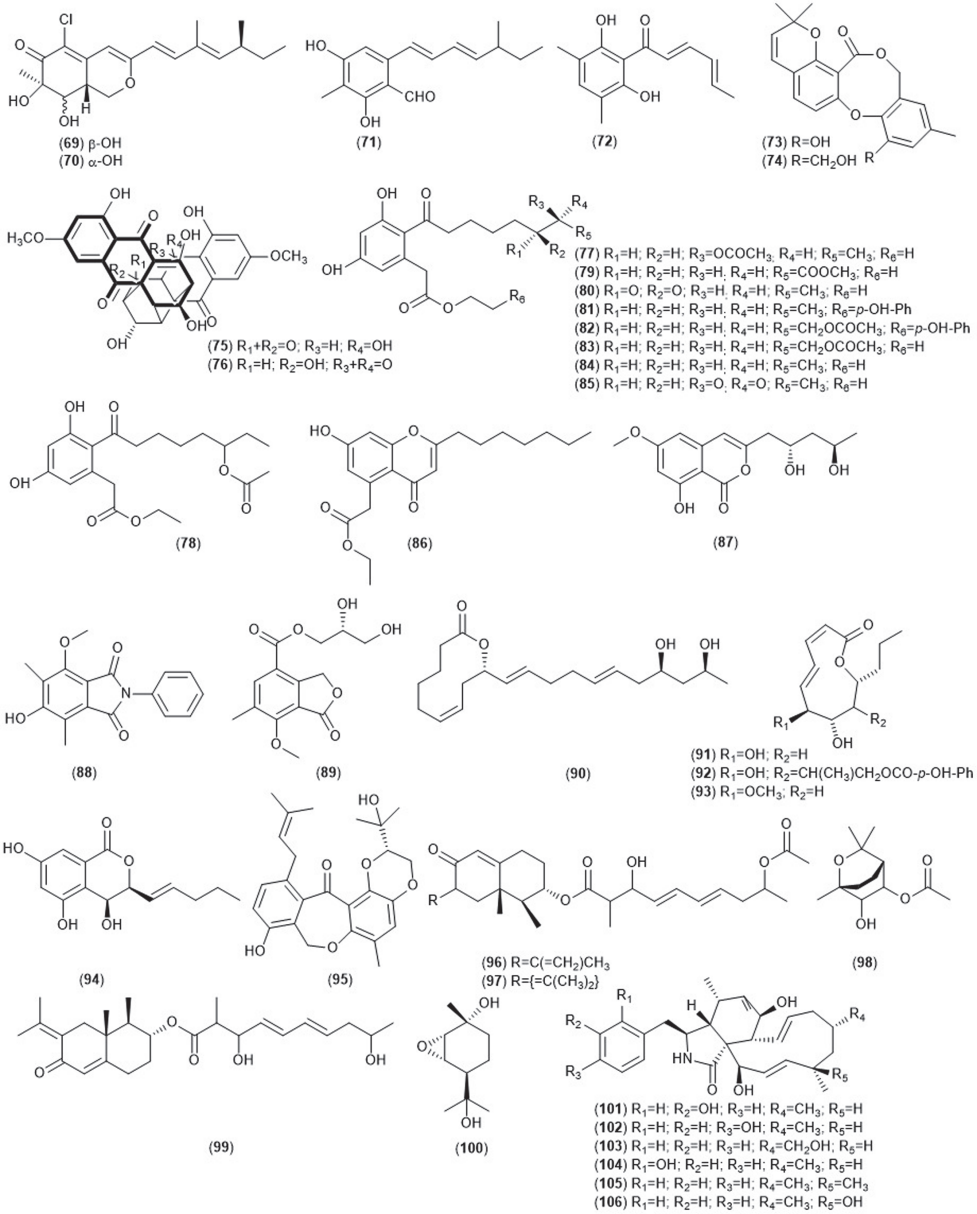
Bioactive compounds (69-106) isolated from endophytic *Diaporthe* sp.

**Fig. 1D:**
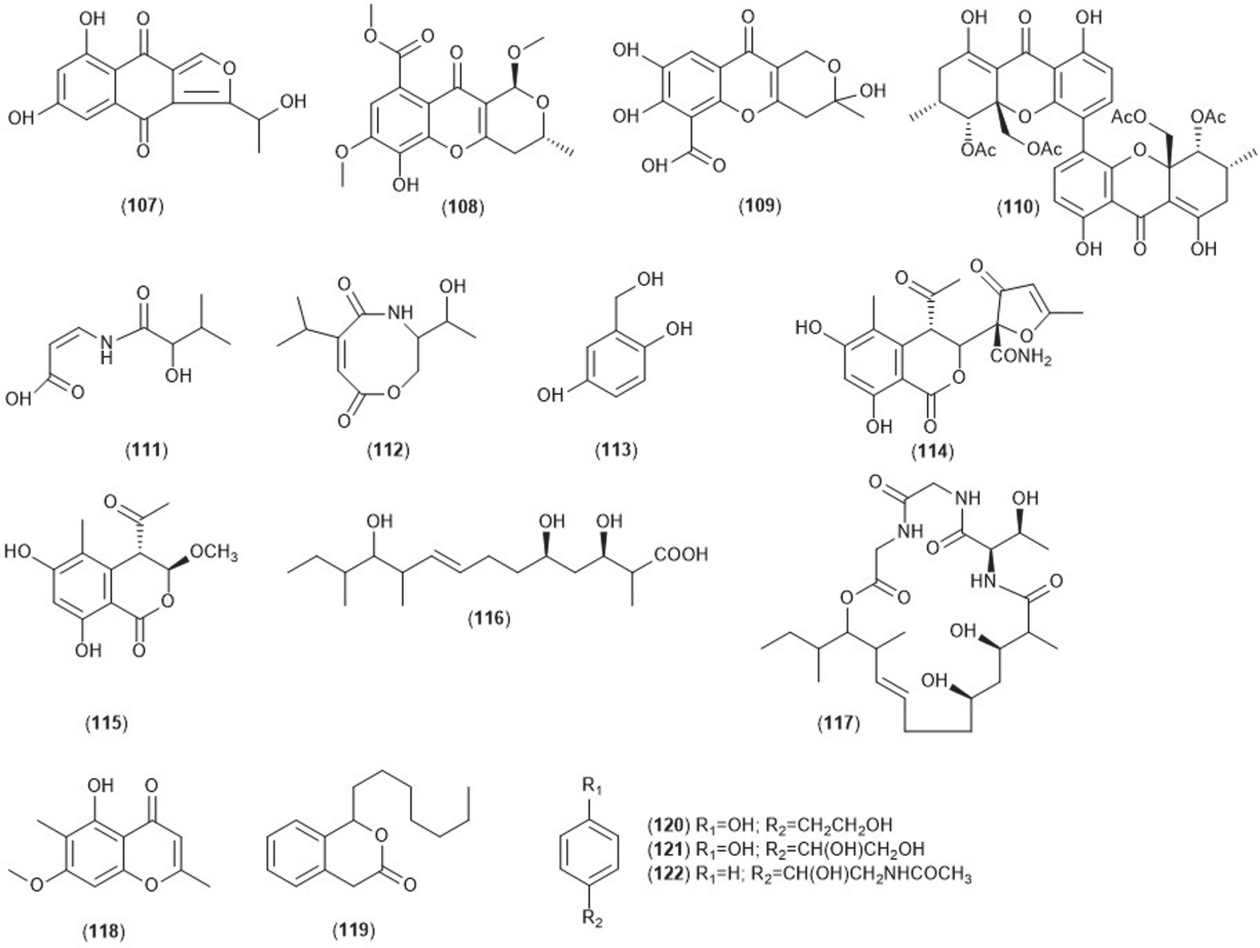
Bioactive compounds (107-122) isolated from endophytic *Diaporthe* sp.

**Table 1:** Source of diverse bioactive compounds isolated from endophytic *Diaporthe* sp. and their potential biological activities.

*Diaporthe* sp.	Host plant	Compounds	Chemical nature	Activity	Cell/Target	Ref.
*Diaporthe* sp. SNB-GSS10	*Sabicea cinerea*	Mycoepoxydiene (**1**); Altiloxin A (**2**); Enamidin (**3**); Eremofortin F (**4**)	Mycoepoxidiene; Terpenoids; Enamidines; Sesquiterpenoids	Cytotoxic	KB (human uterine cervical carcinoma cell); MRC-5 (human lung fibroblast cell); MDA-MB-435 (human breast cancer cell)	[7]
*Diaporthe* sp. LG23	*Mahonia fortunei*	19-*nor*-Lanosta-5(10),6,8,24-tetraene-1*α*,3*β*,12*β*,22S-tetraol (**5**); 3*β*,5*α*,9*α*-Trihydroxy-(22*E*,24*R*)-ergosta-7,22-dien-6-one (**6**); 3*β*,5*α*,9*α*,14*α*-Tetrahydroxy-(22*E*,24*R*)-ergosta-7,22-dien-6-one (**7**); (22*E*,24*R*)-Ergosta-7,9(11),22-triene-3*β*,5*α*,6*α*-triol (**8**); Chaxine C (**9**); Demethylincisterol A_3_ (**10**); Volemolide (**11**)	Triterpenoids	Antibacterial	*Staphylococcus aureus* (Gram-positive)*; Escherichia coli* (Gram-negative)*; Bacillus subtilis* (Gram-positive)*; Pseudomonas aeruginosa* (Gram-negative)*; Streptococcus pyogenes* (Gram-positive)	[23]
*Diaporthe* sp. 1308-05	*Aucuba japonica* var. *borealis*	Homopetasinic acid (**12**)	Acid derivatives	Cytotoxic	*Cochliobolus miyabeanus* (fungus); COLO 201 (human colon adenocarcinomacell); HL60 (human promyelocytic leukemia cell)	[25]
*Diaporthe citri* G-01	*Mikania glomerata* Spreng	3-Nitropropionic acid (**13**)	Acid derivatives	Sub-lethal	Larvae of *Diatraea saccharalis* (sugarcane borer)	[2]
*Diaporthe maritima* DAOMC628553	*Picea rubens*	Phomopsolides A-C (**14**-**16**); (*S*,*E* )-6-(4-hydroxy-3-oxopent-1-en-1-yl)-2*H*-pyran-2-one (**17**)	Pyranones	Antibiotic	*Bacillus subtilis* (Gram-positive)	[6]
*Phomopsis* sp. PSU-H188	*Hevea brasiliensis*	Phomopsisporones A-B (**18**-**19**); (*E*)-1893A (**20**); 1893B (**21**); Mycoepoxydiene (**1**); 2,3-Dihydromycoepoxydiene (**22**); Deacetylmycoepoxydiene (**23**); Nectriapyrone (**24**); Cytochalasin N (**25**); Diaporthalasin (**26**); (3*R*)-5-Methylmellein (**27**); (3*R*,4*R*)-*cis*-4-Hydroxy-5-methylmellein (**28**); (*R*)-(−)-5-Hydroxymethylmellein (**29**); Dothiorelones A-C and E (**30**-**33**); Cytosporones B and D (34-35)	Cytosporones; Mycoepoxydienes; Pyranones; Cytochalasins; Melleins; Pyridine derivatives	Antibacterial; Cytotoxic	*Staphylococcus aureus* (Gram-positive); MRSA (Gram-positive); KB (human oral cavity cancer cell); MCF-7 (human breast cancer cell); Vero (kidney fibroblast cells)	[12]
*Diaporthe phaseolorum* SKS019	*Acanthus ilicifolius*	Diaporphasines A-D (**36**-**39**); Meyeroguillines A, C and D (**40**-**42**); 5-Deoxybostrycoidin (**43**); Fusaristatin A (**44**)	Alkaloids	Cytotoxic	MDA-MB-435 (human breast cancer cell); HepG2 (human liver cancer cell); HCT116 (human colon cancer cell); NCI-H460 (human non-small cell lung cancer cell); MCF10A (human normal breast cell)	[21]
*Diaporthe arengae* TATW2	*Terminalia arjuna* (Roxb.)	Methyl 3-(3,5-di-tert-butyl-4-hydroxyphenyl)propionate (**45**); Pterin-6-carboxylic acid (**46**); 2,6-Ditert-butyl-4-phenol (**47**)	Phenolics	Anti-hypercholesterolemic	hRBC (red blood cell)	[1]
*Diaporthe toxica*	*Lupinus* sp.	Phomopsin F (**48**)	Hexapeptides	Cytotoxic	HepG2 (human liver cancer cell)	[26]
*Diaporthe sp.* SYSU-HQ3	*Excoecaria agallocha*	Diaporisoindoles A-E (**49**-**53**); Tenellones C and D (**54**-**55**); Diaporindenes A-D (**56**-**59**); Isoprenylisobenzofuran A (**60**)	Alkaloids; Benzophenones; Alkaloids	Anti-tuberculosis; LPS-activated NO production	MptpB (*Mycobacterium tuberculosis* protein tyrosine phosphatase B); RAW 264.7 (murine macrophage cell)	[27] [28]
*Diaporthe* sp. SCSIO 41011	*Rhizophora stylosa*	Isochromophilones A-F (**61**-**66**); 5-Chloroisorotiorin (**67**); *epi*-Isochromophilone (**68**); Isochromophilone III (**69**); *epi*-Isochromophilone III (**70**); 6-((1*E*,3*E*)-3,5-Dimethylhepta-1,3-dien-1-yl)-2,4-dihydroxy-3-methylbenzaldehyde (**71**); (2*E*,4*E*)-1-(2,6-Dihydroxy-3,5-dimethylphenyl)hexa-2,4-dien-1-one (**72**)	Azaphilone derivatives	Cytotoxic	ACHN; OS-RC-2; 786-O cells (human renal cancer cells)	[20]
*Diaporthe* sp. ECN-137	*Phellodendron amurense*	Diaporthols A and B (**73**-**74**)	Polyketides	Anti-migration	TGF-*β*1-elicited MDA-MB-231 (human breast cancer cell)	[16]
*Diaporthe* sp. ARL-09	*Anoectochilus roxburghii*	Cytoskyrin C (**75**); Epicytoskyrin (**76**)	Bisanthraquinones	Cytotoxic	SMMC-7721 (human hepatoma cell)	[29]
*Diaporthe pseudomangiferaea*	*Tylophora ouata*	Acetoxydothiorelones A and B (**77**-**78**); Dothiorelones K-N (**79**-**82**); 16-Acetoxydothiorelone C (**83**); Dothiorelones A-C (**30**-**32**); Dothiorelones G and I (**84**-**85**); Cytosporone D (**35**); Pestalotiopsone B (**86**); Mucorisocoumarin A (**87**); 5-Hydroxy-7-methoxy-4,6-dimethyl-2-phenylisoindoline-1,3-dione (**88**); Diaporphthalide A (**89**); Diaporlactone A (**90**)	Thiorelone	Anti-ﬁbrosis; Cytotoxicity; Anti-diabetic	MRC-5 (human lung fibroblasts cell); BGC-823 (human gastric carcinoma cell); PTP1B (protein tyrosine phosphatase 1B)	[30]
*Diaporthe* sp.	*Datura inoxia* Mill	Xylarolide (**91**); Xylarolides A and B (**92**-**93**); Diportharine A (**94**)	Nonenolides; Coumarins	Antioxidant; Cytotoxic	DPPH; MIAPaCa-2 (human pancreatic cancer cell); PC-3 (human prostate cancer cell)	[24]
*Diaporthe lithocarpus* A740	*Morinda oﬃcinalis*	Tenllone I (**95**); Lithocarins B-D (**96**-**98**); Tenellone H (**99**); Phomopene (**100**)	Benzophenones; Eremophilanes; Monoterpentoids	Cytotoxic	SF-268 (human glioblastoma cell); MCF-7 (human breast cancer cell); HepG2 (human liver cancer cell); A549 (human lung adenocarcinoma cell)	[8]
*Diaporthe* sp. GZU-1021	*Chiromanteshaematochir*	Diaporthichalasins A-C (**101**-**103**); Phomopsichalasin G (**104**); 21-*O*-Deacetyl-L-696,474 (**105**); Cytochalasin H (**106**); Biatriosporin N (**107**); Phomopsichin B (**108**); Penialidin A (**109**); Phomoxanthone A (**110**)	Cytochalasins; Tetrahydroxanthone dimers	LPS-activated NO production; Anti-inflammatory cytotoxic	RAW 264.7 (murine macrophage cell)	[15]
*Diapothe vochysiae* LGMF1583	*Vochysia divergens*	Vochysiamides A and B (**111**-**112**); 2,5-Dihydroxybenzyl alcohol (**113**)	Carboxiamides; Alcohols	Antibacterial; Cytotoxic	MSSA (Gram-positive); MRSA (Gram-positive); *Klebsiella. pneumonia* (Gram-negative)*;* A549 (human lung adenocarcinoma cell); PC-3 (human prostate cancer cell)	[5]
*Phomopsis sp.* CFS42	*Cephalotaxus fortunei* Hook	Phomotide A (**114**); 4-Acetyl-3,4-dihydro-6,8-dihydroxy- 3-methoxy-5-methylisocoumarin (**115**)	Polyketides	-	-	[9]
*Diaporthe eucalyptorum* KY-9	*Melia azedarach* Linn	Eucalyptacid A (**116**); Eucalactam B (**117**); Eugenitol (**118**); Cytosporone C (**119**); 4-Hydroxyphenethyl alcohol (**120**); 1-(4-Hydroxyphenyl)ethane-1,2-diol (**121**); *N*-(2-Hydroxy-2-phenylethyl)acetamide (**122**); Phomopene (**100**)		Antifungal	*Alternaria. solani* (fungus)	[31]
